# Deciphering the Protein, Modular Connections and Precision Medicine for Heart Failure With Preserved Ejection Fraction and Hypertension Based on TMT Quantitative Proteomics and Molecular Docking

**DOI:** 10.3389/fphys.2021.607089

**Published:** 2021-10-14

**Authors:** Guofeng Zhou, Jiye Chen, Chuanhong Wu, Ping Jiang, Yongcheng Wang, Yongjian Zhang, Yuehua Jiang, Xiao Li

**Affiliations:** ^1^First Clinical Medical College, Shandong University of Traditional Chinese Medicine, Jinan, China; ^2^The Biomedical Sciences Institute of Qingdao University (Qingdao Branch of SJTU Bio-X Institutes), Qingdao University, Qingdao, China; ^3^Affiliated Hospital of Shandong University of Traditional Chinese Medicine, Jinan, China

**Keywords:** hypertension, heart failure with preserved ejection fraction, molecular docking, modular, therapeutic prediction

## Abstract

**Background:** Exploring the potential biological relationships between heart failure with preserved ejection fraction (HFpEF) and concomitant diseases has been the focus of many studies for the establishment of personalized therapies. Hypertension (HTN) is the most common concomitant disease in HFpEF patients, but the functional connections between HFpEF and HTN are still not fully understood and effective treatment strategies are still lacking.

**Methods:** In this study, tandem mass tag (TMT) quantitative proteomics was used to identify disease-related proteins and construct disease-related networks. Furthermore, functional enrichment analysis of overlapping network modules was used to determine the functional similarities between HFpEF and HTN. Molecular docking and module analyses were combined to identify therapeutic targets for HFpEF and HTN.

**Results:** Seven common differentially expressed proteins (co-DEPs) and eight overlapping modules were identified in HFpEF and HTN. The common biological processes between HFpEF and HTN were mainly related to energy metabolism. Myocardial contraction, energy metabolism, apoptosis, oxidative stress, immune response, and cardiac hypertrophy were all closely associated with HFpEF and HTN. Epinephrine, sulfadimethoxine, chloroform, and prednisolone acetate were best matched with the co-DEPs by molecular docking analyses.

**Conclusion:** Myocardial contraction, energy metabolism, apoptosis, oxidative stress, immune response, and cardiac hypertrophy were the main functional connections between HFpEF and HTN. Epinephrine, sulfadimethoxine, chloroform, and prednisolone acetate could potentially be effective for the treatment of HTN and HFpEF.

## Introduction

Heart failure with preserved ejection fraction (HFpEF), which is a complex syndrome characterized by a normal left ventricular ejection fraction and abnormal diastolic function, accounts for more than 50% of heart failure (HF) patients (Pieske et al., 2019). Current therapies for HFpEF include strategies to manage the coexisting conditions, reduce symptoms, and treat volume overload when necessary (Redfield, 2016). Although there has been much progress in HFpEF-related research, an effective strategy for HFpEF treatment has not yet been established (Gazewood and Turner, 2017). Compared with heart failure with reduced ejection fraction (HFrEF), HFpEF is heterogeneous, and drugs effective against HFrEF are not suitable for HFpEF (Graziani et al., 2018). Thus, a complete clinical phenotypic classification of HFpEF, including tiology, concomitant diseases, and risk factors, is required. Furthermore, exploring the underlying biological functions involved in the different types of HFpEF will help develop personalized therapies and precision medicines for HFpEF (Borlaug, 2020; Ge, 2020).

Patients with HFpEF that are diagnosed with hypertension (HTN) and coronary heart disease are regarded as having vascular-related HFpEF (Ge, 2020). Studies have shown that HTN is the most common complication in patients with HFpEF, but the biological relationship between HTN and HFpEF is still not fully understood (Tadic et al., 2018). In addition, studies have suggested that HTN is an additional risk factor for HFpEF (Dunlay et al., 2017). HFpEF and HTN share many common pathogeneses, such as dysfunction of cardiac autonomy, imbalance of the renin-angiotensin-aldosterone system, and excessive oxidative stress. In addition, some underlying biological mechanisms play an important role in the transition from HTN to HFpEF. For example, hypertension leads to diastolic dysfunction and concentric remodeled left ventricular decompensation, resulting in HFpEF (Drazner, 2011; Heinzel et al., 2015; Messerli et al., 2017; Nwabuo and Vasan, 2020). In addition, HTN also activates chronic inflammation and increases collagen deposition, further exacerbating left ventricular dysfunction (Paulus and Tschöpe, 2013). Studies have reported that myocardial contractile dysfunction, right ventricular dysfunction, arterial stiffness, ventricular-arterial coupling, and microvascular dysfunction could increase the risk of HFpEF in patients with HTN (Hicklin et al., 2020). However, in clinical trials, drugs such as angiotensin-converting enzyme inhibitors, angiotensin II receptor blockers, diuretics, and beta-blockers, which showed beneficial effects against common pathogeneses of HFpEF and HTN, did not produce significant positive effects in patients with HFpEF (Kjeldsen et al., 2020). Therefore, further research is needed to explore the potential biological relationships between HFpEF and HTN.

Disease network construction provides a solution to explore the relationships between diseases (Le and Pham, 2017), where modules of disease-related networks are responsible for various features of the diseases. Functional enrichment analysis of the overlapping modules reflects the functional links among related diseases (Dean et al., 2017). For example, using this method, a previous study showed that the negative regulation of transcription from RNA polymerase II promoter RNA and the negative regulation of apoptotic processes are overlapping biological functions among type-2 diabetes mellitus, prostate cancer, and chronic myeloid leukemia (Liu et al., 2019). Furthermore, another study showed that atherosclerosis, cholesterol homeostasis, plasma lipoprotein particle remodeling, and oxidative stress responses are common risk factors for stroke and coronary heart disease (Zhang et al., 2014).

**FIGURE 1:**
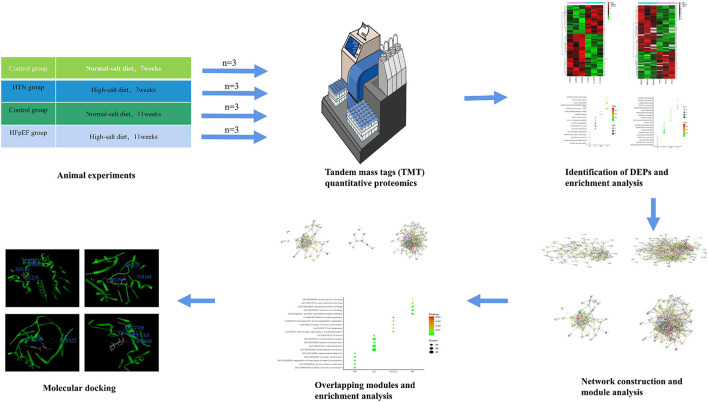
Flowchart of the research design. Deciphering the protein networks and modular connections, and targeting precision medicines for HFpEF and HTN using TMT quantitative proteomics and molecular docking analyses.

The Dahl salt-sensitive (DS) rat model has been implemented for the study of HFpEF (Cho et al., 2017). Toward that, DS rats diagnosed with HTN or HFpEF were analyzed using proteomics. Cytoscape software and STRING platforms were used to construct a disease network. Modules of the disease network were divided using Molecular Complex Detection (MCODE). Gene Ontology (GO) enrichment analysis was performed to identify the significant functions and pathways of overlapping modules found in the Database for Annotation, Visualization, and Integrated Discovery (DAVID). Molecular docking and module analyses were combined to contribute to the development of personalized therapies and precision medicines for HFpEF and HTN treatment. A flowchart of the research design is shown in Figure 1.

## Materials and Methods

### Animals and Experimental Protocols

Specific pathogen-free 6-week-old male DS rats (weight: 160–180 g; Certificate No. 2016-0006) were obtained from the Charles River Animal Laboratory (Beijing, China). Rats were housed in groups of six rats per cage under controlled conditions (12 h dark/light cycle, temperature: 20–24°C, relative humidity: 40–60%, dB ≤ 60) and with free access to water and food. After a week of adaptation, the rats were randomly divided into the following three groups: the HTN group (8% NaCl chow for 7 weeks, *n* = 6), the HFpEF group (8% NaCl chow for 11 weeks, *n* = 6), and the control group (0.3% NaCl chow for 7 or 11 weeks, *n* = 12). All experiments were reviewed by the Animal Ethics Committee of the Shandong University of Traditional Chinese Medicine (Ethics No. SDUTCM2018071501).

### Tissue Collection

The rats in the HTN and HFpEF groups were euthanized after 7 and 11 weeks of the high-salt diet (HSD), respectively, and the rats in the control group were randomly sacrificed after 7 or 11 weeks of the control diet. Pentobarbital (20 mg/kg, i.p.) was used for anesthesia in rats, and the left ventricle (LV) was collected from each rat and stored at −80°C.

### Tandem Mass Tag-Labeled Quantitative Proteomics

Twelve LV samples [HTN group *n* = 3, HFpEF group *n* = 3, control group (euthanized at 7 weeks) *n* = 3, control group (euthanized at 11 weeks) *n* = 3] were collected for TMT quantitative proteomics. Previous studies reported that data with three samples in each group could reliably be statistically analyzed (Maitiabola et al., 2020; Yan et al., 2020). The tissue was removed from the refrigerator at −80°C, ground into powder, and quickly transferred to a centrifuge tube pre-cooled with liquid nitrogen. PASP protein lysate (100 mM ammonium bicarbonate, 8 M urea, pH 8) was added to the liquid nitrogen, shaken, mixed, and ultrasonicated in an ice water bath for 5 min, followed by centrifugation at 12,000 rpm for 15 min at 4°C. Then, the supernatant was collected, 10 nM dithiothreitol was added, and the mixture was incubated at 56°C for 1 h. Then, iodoacetamide was added and the reaction was allowed to proceed for 1 h in the absence of light. Next, four volumes of pre-cooled acetone were used for precipitation, followed by centrifugation at 12,000 rpm for 15 min at 4°C, after which the precipitate was collected. The precipitate was resuspended and washed with one milliliter of −20°C pre-cooled acetone, followed by a second centrifugation at 12,000 rpm for 15 min at 4°C. Then, the precipitate was collected and air dried, and an appropriate amount of protein dissolving solution (8 M urea, 100 mM TEAB, pH 8.5) was used to dissolve the protein precipitate.

The Bradford protein quantification kit (Beyotime, China) was used to determine the protein concentration. DB protein dissolving solution (8 M urea, 100 mM TEAB, pH 8.5) was added to the protein sample to a volume of 100 μL, trypsin and 100 mM buffer were added, and mixing and digestion were performed at 37°C for 4 h. Then, pancreatin and CaCl_2_ were used for digestion overnight. Formic acid was used to adjust the pH to less than 3, mixing was done at room temperature, and centrifugation was performed at 12,000 rpm for 5 min. The supernatant was then slowly passed through the C18 desalting column, and the cleaning solution (0.1% formic acid, 3% acetonitrile) was used for washing three times. In addition, an appropriate amount of eluent (0.1% formic acid, 70% acetonitrile) was added, and the filtrate was collected and lyophilized. One hundred microliters of 0.1 M TEAB buffer was used for reconstitution, and 41 μL of TMT labeling reagent was dissolved in acetonitrile. The mixture was mixed at room temperature for 2 h, and 8% ammonia was added to stop the reaction. An equal volume of the labeled sample was used for mixing and freeze-drying after desalting.

Mobile phase A solution (2% acetonitrile, 98% water, pH 10) and mobile phase B solution (98% acetonitrile, 2% water) were prepared. The freeze-dried powder was dissolved in solution A and centrifuged at 12,000 rpm for 10 min at room temperature. An L-3000 HPLC system and a water VEHC 18 (4.6 mm × 250 mm, 5 μm) were used for this study, and the column temperature was set to 45°C. Details of the elution gradient are shown in Table 1. One tube was collected every minute, divided into 10 fractions, freeze-dried, and dissolved in 0.1% formic acid.

**TABLE 1:** The elution gradient table of peptide fraction separation liquid chromatography.

Time (min)	Flow rate (mL/min)	Mobile phase A (%)	Mobile phase B (%)
0	1	97	3
10	1	95	5
30	1	80	20
48	1	60	40
50	1	50	50
53	1	30	70
54	1	0	100

Mobile phase A solution (100% water, 0.1% formic acid) and phase B solution (80% acetonitrile, 0.1% formic acid) were prepared. One microgram of the supernatant from each fraction was used for the test. The UHPLC system was upgraded with the EASY-nLC 1200 system. We prepared both the pre-column (4.5 cm × 75 μm, 3 μm) and the analytical column (15 cm × 150 μm, 1.9 μm). The elution conditions for liquid chromatography are shown in Table 2. A Q Exactive series mass spectrometer was used for this study, the ion source was the Nanospray Flex, the ion spray voltage was 2.3 kV, the temperature of the ion transfer tube was 320°C, and the data-dependent acquisition mode was used. The full scan range of the mass spectrum was 350–1,500 m/z. The resolution of the primary mass spectrometry was set to 60,000 (200 m/z), the maximum capacity of the C-trap was 3 × 10^6^, and the maximum injection time of the C-trap was 20 ms. The top 40 precursor ions were selected for the full scan, and the higher-energy collision dissociation (HCD) method was used for the fragment, which contributed to the secondary mass spectrometry detection. The isolation window of MS2 spectrum is 2 m/z. HCD spectrum ranged from 120 to m/z (precursor ion) × z (charge number) + 100 m/z. The resolution was 45,000 (200 m/z), the maximum capacity of the C-trap was 5 × 10^4^, the maximum injection time of the C-trap was 86 ms, the threshold intensity was 1.2 × 10^5^, and the dynamic exclusion range was 20 s.

**TABLE 2:** Elution gradient table of liquid chromatography.

Time (min)	Flow rate (nL/min)	Mobile phase A (%)	Mobile phase B (%)
0	600	94	6
2	600	85	15
78.5	600	60	40
80.5	600	50	50
81.5	600	45	55
90	600	0	100

MS/MS raw files were processed using the MASCOT engine (Matrix Science, London, United Kingdom; version 2.6) embedded into Proteome Discoverer software, and searched against the UniProt database, including Uniprot_RattusNorvegicus_36080_20180123 sequences^1^. The search parameters included trypsin as the enzyme used to generate peptides with a maximum of two missed cleavages permitted. A precursor mass tolerance of 10 ppm was specified along with a 0.05 Da tolerance for MS2 fragments. Except for the TMT labels, carbamidomethyl (C) was set as a fixed modification. The variable modifications were oxidation (M) and acetyl (protein N-term). A peptide and protein false discovery rate of 1% was enforced using a reverse database search strategy. The quantitative values of proteins obtained from two pairs of samples were examined using the *t*-test, and the *p*-values were calculated. Fold change >1.1, fold change <0.91, and *P*-value < 0.05, were considered to filter differentially expressed proteins (DEPs). Proteomic data is provided as Supplementary Material.

### Constructing the Protein-Protein Interaction Networks for Heart Failure With Preserved Ejection Fraction and Hypertension

The STRING database (version 10.5)^2^ was used to predict protein interactions and functional associations. The PPI networks of HFpEF- and HTN-DEPs were obtained under controlled parameters (interaction score >0.4). PPI networks were analyzed using Cytoscape (version 3.6.1)^3^.

### Functional Enrichment Analysis

HFpEF- and HTN-DEPs were submitted to the Database for annotation, visualization, and integrated discovery for functional enrichment, including GO and Kyoto Encyclopedia of Genes and Genomes (KEGG) pathway enrichment analyses (version 6.8)^4^. The *P*-value was set at <0.05 for GO and KEGG pathway enrichment, as is standard in the field (Yuan et al., 2016; Liu et al., 2019).

### Division and Identification of Network Modules

Disease-related networks were analyzed using Cytoscape (version 3.6.1)^5^. Furthermore, the modules were divided by Molecular Complex Detection (MCODE, version 1.3.2)^6^. The modules were obtained under controlled parameters (degree cutoff = 2, node score cutoff = 0.1, core threshold *K* = 2, flux density cutoff = 0.1, K-core 2, max. depth = 100). The parameters for Cytoscape were set as default, as recommended by previous studies (Yuan et al., 2016; Liu et al., 2019).

### Identification of Modern Medicine Symptoms Related to Common Differentially Expressed Proteins and Links to Cardiovascular Diseases

The association between the co-DEPs and cardiovascular diseases was analyzed using the Comparative Toxicogenomics Database (CTD)^7^, which integrates the relationships between gene products, diseases, chemicals, and environments. Furthermore, related MM symptoms of the co-DEPs were observed in SymMap^8^ and in previous publications.

### Drug Discovery and Molecular Docking

Small-molecule compounds related to the co-DEPs were observed from the DrugBank^9^, through the target search. Molecular docking analysis is a common strategy for drug discovery. The structures of the co-DEPs were downloaded from the Protein Data Bank (PDB)^10^, and the PDB IDs of the proteins are shown in Table 3. The structures of the drugs were obtained from PubChem^11^, and the CIDs are shown in Table 4. Eutectic ligands and water molecules were removed, and residue repair, side chain fixation, and hydrogenation were used for protein preparation. The Surflex-Dock module of SYBYL 2.1 was used for molecular docking. Furthermore, AMBR7 was used for energy optimization, and the active pockets were obtained in automatic mode. The parameters for SYBYL 2.1 were set as default, as recommended by previous studies (Tan et al., 2020). The molecular docking scores reflected the binding effects between the small-molecule compounds and the co-DEPs, and the highest scoring small molecules were considered as potential drugs.

**TABLE 3:** PDB ID of protein.

Protein	PDB ID
Hp	4X0lL
COQ9	6awl
Tf	1ryo
Prnp	2ol9
Acta1	2ib8
Timm44	2cw9
Abcb6	3nh6

**TABLE 4:** CIDs of molecule compounds.

Molecule	PubChem ID
Prednisolone acetate	5834
Bismuth subsalicylate	16682734
Phenoxymethylpenicillin	6869
Polyethylene glycol	40786
Prednisolone	5755
Chloroform	6212
Salicylic acid	338
Epinephrine	5816
Triptorelin	25074470
Benzylpenicillin	5904
Propofol	4743
Sulfadimethoxine	5323

## Results

### Identification of Differentially Expressed Proteins

**FIGURE 2:**
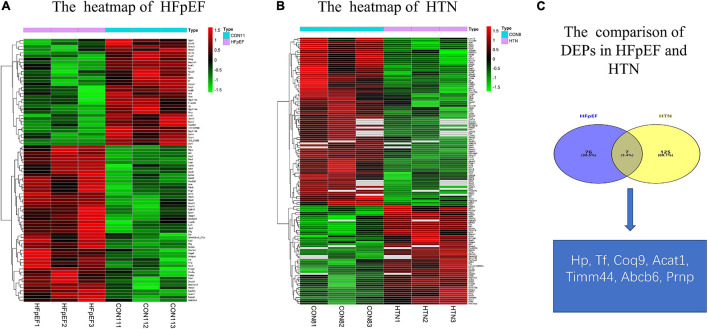
Comparison of the DEPs in HFpEF and HTN. **(A)** Heatmap of HFpEF. **(B)** Heatmap of HTN. **(C)** The co-DEPs between HFpEF and HTN. Hp, haptoglobin; COQ9, coenzyme Q9; Tf, serotransferrin; Prnp, major prion protein; Acat1, acetyl-CoA acetyltransferase, mitochondrial; Timm44, translocase of inner mitochondrial membrane 44; Abcb6, ATP-binding cassette sub-family B member 6.

**FIGURE 3:**
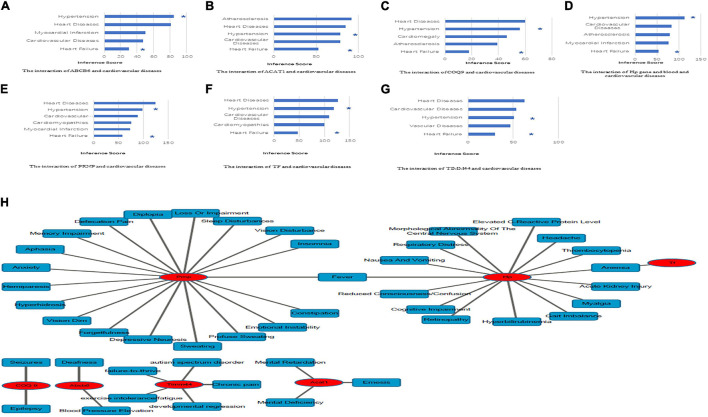
Identification of co-DEP-related MM symptoms and links to cardiovascular diseases. **(A)** Abcb6, **(B)** Acat1, **(C)** COQ9, **(D)** Hp, **(E)** Prnp, **(F)** Tf, and **(G)** Timm44. *, direct evidence. **(H)** The related MM symptoms of the co-DEPs. Hp, haptoglobin; COQ9, coenzyme Q9; Tf, serotransferrin; Prnp, Major prion protein; Acat1, acetyl-CoA acetyltransferase, mitochondrial; Timm44, translocase of inner mitochondrial membrane 44; Abcb6, ATP-binding cassette sub-family B member 6.

A total of 83 DEPs, including 52 upregulated proteins, were obtained by comparing the HFpEF group with the control group sacrificed at 11 weeks (Figure 2A). A total of 132 DEPs, including 85 upregulated proteins, were identified by comparing the HTN group with the control group sacrificed at 7 weeks (Figure 2B). Among the HFpEF-DEPs and HTN-DEPs, there were 7 co-DEPs, including haptoglobin (Hp), coenzyme Q9 (COQ9), serotransferrin (Tf), major prion protein (Prnp), acetyl-CoA acetyltransferase, mitochondrial (Acat1), translocase of inner mitochondrial membrane 44 (Timm44), and ATP-binding cassette sub-family B member 6 (Abcb6; Figure 2C). Furthermore, the CTD database showed links between the co-DEPs and various cardiovascular diseases (Figures 3A–G). Finally, the related MM symptoms of the co-DEPs were determined using SymMap^12^ and previous publications (Figure 3H).

### Functional Enrichment Analysis

**FIGURE 4:**
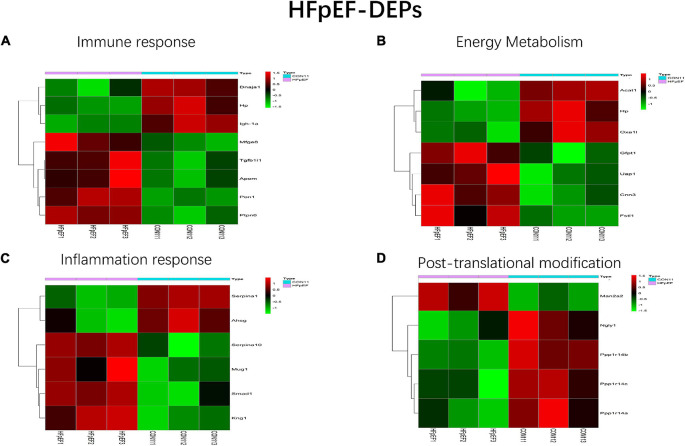
Main biological processes enriched in HFpEF-DEPs. **(A)** Immune response, **(B)** energy metabolism, **(C)** inflammation response, and **(D)** post-translational modification.

**FIGURE 5:**
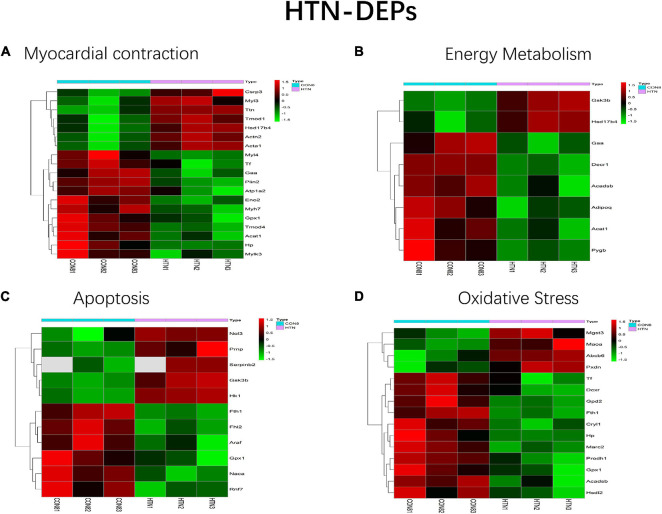
Main biological processes enriched in HTN-DEPs. **(A)** Myocardial contraction, **(B)** energy metabolism, **(C)** apoptosis, and **(D)** oxidative stress.

All HFpEF- and HTN-DEPs were submitted to DAVID for GO and KEGG functional enrichment analyses. The DEPs of HFpEF were mainly involved in immune response, energy metabolism, inflammation response, and post-translational modification (Figure 4). For example, DnaJ heat shock protein family (Hsp40) member A1 (Dnaja1), Hp, immunoglobulin heavy chain 6 (Igh-1a), milk fat globule EGF and factor V/VIII domain containing (Mfge8), transforming growth factor beta 1 induced transcript 1 (Tgfb1i1), apolipoprotein M (Apom), paraoxonase 1 (Pon1), protein tyrosine phosphatase, and non-receptor type 6 (Ptpn6) were closely related to immune response; acetyl-CoA acetyltransferase, mitochondrial (Acta1), Hp, mitochondrial inner membrane protein (Oxa1l), glutamine fructose-6-phosphate transaminase 1 (Gfpt1), UDP-N-acetylglucosamine pyrophosphorylase 1 (Uap1), calponin 3 (Cnn3), and follistatin-like 1 (Fstl1) were involved in energy metabolism; serpin family A member 1 (Serpina1), alpha-2-HS-glycoprotein (Ahsg), serpin family A member 10 (Serpina10), murinoglobulin 1 (Mug1), SMAD family member 1 (Smad1), and kininogen 1 (Kng1) were connected with inflammation response; mannosidase, alpha, class 2A, member 2 (Man2a2), N-glycanase 1 (Ngly1), protein phosphatase 1, regulatory (inhibitor) subunit 14B (Ppp1r14b), protein phosphatase 1, regulatory (inhibitor) subunit 14C (Ppp1r14c), protein phosphatase 1, and regulatory (inhibitor) subunit 14A (Ppp1r14a) were involved in post-translational modification. For HTN, myocardial contraction, energy metabolism, apoptosis, and oxidative stress were the main biological functions (Figure 5). Carcass protein in high growth mice 3 (Carp3), myosin light chain 3 (Myl3), titin (Ttn), tropomodulin (Tmod1), hydroxysteroid (17-beta) dehydrogenase 4 (Hsd17b4), actinin alpha 2 (Actn2), Acta1, myosin, light chain 4 (Myl4), Tf, alpha glucosidase (Gaa), perilipin 2 (Plin2), ATPase Na+/K+ transporting subunit alpha 2 (Atp1a2), enolase 2 (Eno2), myosin heavy chain 7 (Myh7), glutathione peroxidase 1 (Gpx1), tropomodulin 4 (Tmod4), Acta1, Hp, and myosin light chain kinase 3 (Mylk3) were closely related to myocardial contraction; glycogen synthase kinase 3 beta (Gsk3b), hydroxysteroid (17-beta) dehydrogenase 4 (Hsd17b4), alpha glucosidase (Gaa), 2,4-dienoyl-CoA reductase 1 (Decr1), acyl-CoA dehydrogenase, short/branched chain (Acadsb), adiponectin C1Q and collagen domain containing (Adipoq), Acta1, and glycogen phosphorylase B (Pygb) were involved in energy metabolism; nucleolar protein 3 (Nol3), prion protein (Prnp), serpin family B member 2 (Serpinb2), glycogen synthase kinase 3 beta (Gsk3b), hexokinase 1 (Hk1), ferritin heavy chain 1 (Fth1), four and a half LIM domains 2 (Fhl2), A-Raf proto-oncogene, serine/threonine kinase (Araf), glutathione peroxidase 1 (Gpx1), nascent polypeptide associated complex subunit alpha (Naca), and ring finger protein 7 (Rnf7) were connected with apoptosis.

**FIGURE 6:**
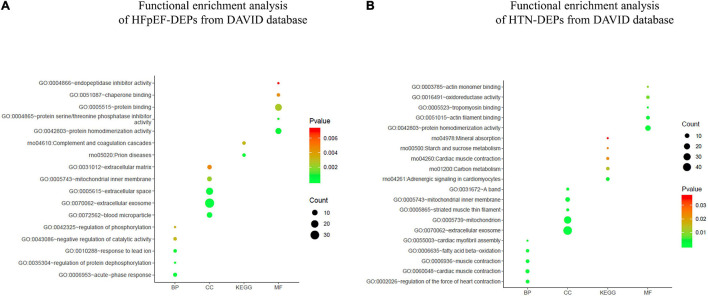
Functional enrichment analysis. **(A)** Functional enrichment analysis of HFpEF-DEPs from the DAVID database. **(B)** Functional enrichment analysis of HTN-DEPs from the DAVID database.

The top five biological processes among HFpEF-DEPs were acute-phase response (count 5, *P*-Value 2.15E-05), regulation of protein dephosphorylation (count 3, *P*-Value 2.75E-04), response to lead ion (count 4, *P*-Value 6.39E-04), negative regulation of catalytic activity (count 4, *P*-Value 0.003203462), and regulation of phosphorylation (count 3, *P*-Value 0.003348677). Blood microparticles (count 10, *P*-Value 1.80E-09), extracellular exosome (count 33, *P*-Value 1.01E-08), extracellular space (count 18, *P*-Value 3.18E-05), mitochondrial inner membrane (count 7, *P*-Value 0.002195356), and extracellular matrix (count 6, *P*-Value 0.00460952) related cell compositions were significantly enriched. Furthermore, the main enriched molecular functions were protein homodimerization activity (count 12, *P*-Value 5.67E-04), protein serine/threonine phosphatase inhibitor activity (count 3, *P*-Value 9.62E-04), protein binding (count 16, *P*-Value 0.002503286), chaperone binding (count 4, *P*-Value 0.004337384), and endopeptidase inhibitor activity (count 3, *P*-Value 0.007219962). The enriched KEGG pathways were prion diseases (count 4, *P*-Value 3.21E-04) and the complement and coagulation cascades (count 4, *P*-Value 0.003144821) (Figure 6A). With respect to HTN-DEPs, the most enriched biological processes were the regulation of heart contraction force (count 6, *P*-Value 2.73E-07), cardiac muscle contraction (count 7, *P*-Value 5.96E-07), muscle contraction (count 6, *P*-Value 1.50E-05), fatty acid beta-oxidation (count 5, *P*-Value 1.95E-04), and cardiac myofibril assembly (count 3, *P*-Value 0.002067005). The main components were extracellular exosome (count 48, *P*-Value 3.12E-11), mitochondrion (count 32, *P*-Value 3.12E-08), striated muscle thin filament (count 4, *P*-Value 3.24E-05), mitochondrial inner membrane (count 10, *P*-Value 2.30E-04), and a band (count 4, *P*-Value 4.49E-04). The molecular functions of HTN-PEGs were mainly enriched in protein homodimerization activity (count 15, *P*-Value 4.74E-04), actin filament binding (count 6, *P*-Value 0.001790743), tropomyosin binding (count 3, *P*-Value 0.003348376), oxidoreductase activity (count 5, *P*-Value 0.00825633), and actin monomer binding (count 3, *P*-Value 0.010556462). The enriched KEGG pathways were adrenergic signaling in cardiomyocytes (count 6, *P*-Value 0.004149388), carbon metabolism (count 5, *P*-Value 0.014082125), cardiac muscle contraction (count 4, *P*-Value 0.022270779), starch and sucrose metabolism (count 3, *P*-Value 0.023739862), and mineral absorption (count 3, *P*-Value 0.03637201) (Figure 6B).

### Protein-Protein Interaction Network Analysis and Modularity Analysis

**FIGURE 7:**
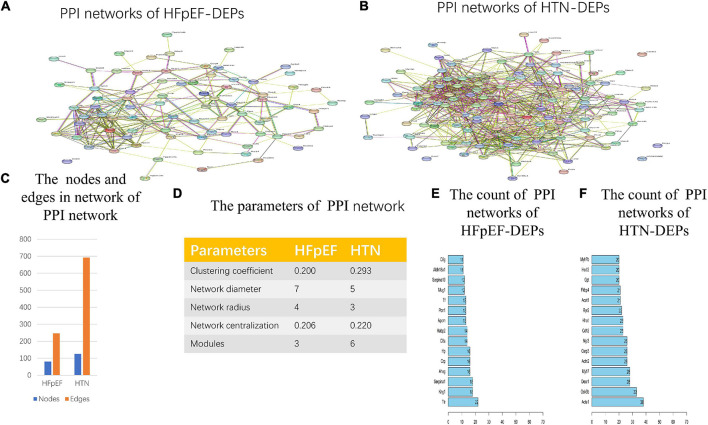
Network analysis of HFpEF and HTN. **(A)** The PPI network of HFpEF-DEPs. **(B)** The PPI network of HTN-DEPs. **(C)** The nodes and edges of the PPI network. **(D)** The parameters of the PPI network. **(E)** The count of the PPI network of HFpEF-DEPs. **(F)** The count of the PPI network of HTN-DEPs.

**FIGURE 8:**
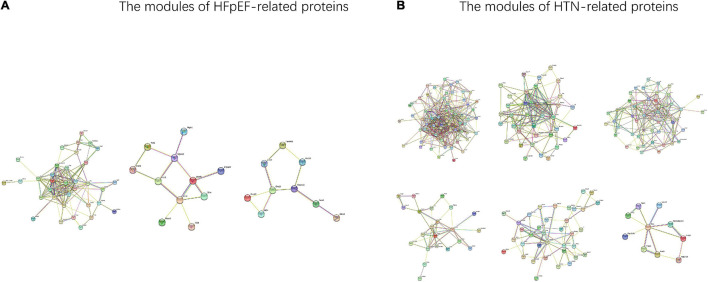
The modules of the HFpEF- and HTN-DEPs. **(A)** The modules of the HFpEF-DEPs. **(B)** The modules of the HTN-DEPs.

In total, from the PPI network of HFpEF-DEPs (Figure 7A) and that of the HTN-DEPs (Figure 7B), 80 nodes and 246 edges, and 126 nodes and 692 edges were identified, respectively (Figure 7C). The parameters of the PPI network of the HFpEF- and HTN-DEPs are shown in Figure 7D. Furthermore, transthyretin (Ttr; degree = 22), kininogen-1 (Kng1; degree = 18), alpha-1-antiproteinase (Serpina1; degree = 18), alpha-2-HS-glycoprotein (Ahsg; degree = 16), and pentaxin (Crp; degree = 16) were identified as hub proteins in the HFpEF-DEP PPI network (Figure 7E). Acetyl-CoA acetyltransferase, mitochondrial (Acta1; degree = 38), glycogen synthase kinase 3 beta (Gsk3b; degree = 33), 2,4-dienoyl-CoA reductase 1 (Decr1; degree = 28), myosin heavy chain 7 (Myh7; degree = 28), and actinin alpha 2 (Actn2; degree = 26) were identified as hub proteins in the HTN-DEP PPI network (Figure 7F). Finally, three modules were obtained from the HFpEF-DEP PPI network (Figure 8A), and five modules were identified from the HTN-DEP PPI network (Figure 8B).

### Overlapping Modules Between the Heart Failure With Preserved Ejection Fraction and Hypertension Networks

**FIGURE 9:**
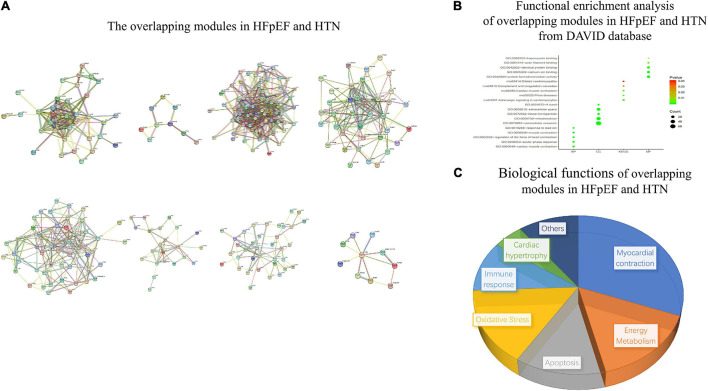
Functional enrichment analysis of overlapping modules between the HFpEF and HTN networks. **(A)** The overlapping modules in HFpEF and HTN. **(B)** Functional enrichment analysis of overlapping modules in HFpEF and HTN from the DAVID database. **(C)** Biological functions of overlapping modules in HFpEF and HTN.

The overlapping modules between the HFpEF and HTN networks are shown in Figure 9A. The top five biological processes among the overlapping modules were cardiac muscle contraction (count 8, *P*-Value 5.59E-08), acute-phase response (count 7, *P*-Value 2.79E-07), regulation of the heart contraction force (count 6, *P*-Value 5.37E-07), muscle contraction (count 6, *P*-Value 2.89E-05), and response to lead ion (count 5, *P*-Value 1.72E-04). Extracellular exosome (count 62, *P*-Value 3.45E-18), mitochondrion (count 33, *P*-Value 1.10E-07), blood microparticle (count 9, *P*-Value 2.42E-06), extracellular space (count 27, *P*-Value 2.68E-06), and a band (count 5, *P*-Value 2.15E-05) related cell compositions were significantly enriched. Furthermore, the main enriched molecular functions were protein homodimerization activity (count 18, *P*-Value 6.42E-05), calcium ion binding (count 15, *P*-Value 4.86E-04), identical protein binding (count 14, *P*-Value 7.87E-04), actin filament binding (count 6, *P*-Value 0.003338211), and tropomyosin binding (count 3, *P*-Value 0.004429531). The enriched KEGG pathways included adrenergic signaling in cardiomyocytes (count 7, *P*-Value 0.001472787), prion diseases (count 4, *P*-Value 0.003172377), cardiac muscle contraction (count 5, *P*-Value 0.005270334), complement and coagulation cascades (count 4, *P*-Value 0.02702271), and adrenergic signaling in cardiomyocytes (count 4, *P*-Value 0.040014194) (Figure 9B). Finally, the main functional biological processes were myocardial contraction (30.77%), energy metabolism (15.38%), apoptosis (12.82%), oxidative stress (15.38%), immune response (10.26%), and cardiac hypertrophy (5.13%) (Figure 9C).

### Drug Discovery and Molecular Docking

**FIGURE 10:**
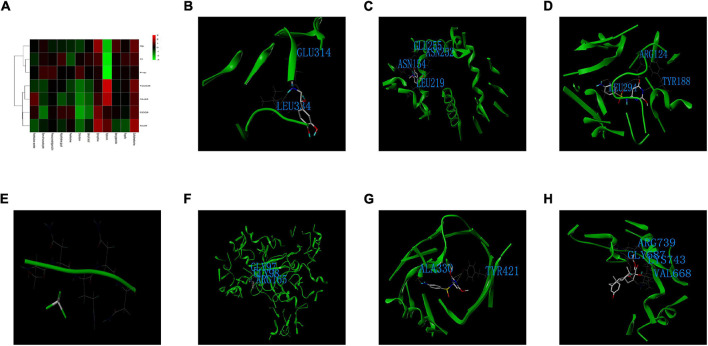
Drug discovery and molecular docking. **(A)** Heatmap of the binding scores between the small-molecule compounds and co-DEPs. **(B)** The binding sites and interactions between epinephrine and Hp. **(C)** The binding sites and interactions between epinephrine and COQ9. **(D)** The binding sites and interactions between sulfadimethoxine and Tf. **(E)** The binding sites and interactions between chloroform and Prnp. **(F)** The binding sites and interactions between epinephrine and Acta1. **(G)** The binding sites and interactions between sulfadimethoxine and Timm44. **(H)** The binding sites and interactions between prednisolone acetate and Abcb6.

Twelve small-molecule compounds were obtained from the DrugBank, including prednisolone acetate, bismuth subsalicylate, phenoxymethylpenicillin, polyethylene glycol, prednisolone, chloroform, salicylic acid, epinephrine, triptorelin, benzylpenicillin, propofol, and sulfadimethoxine. The binding affinity between the co-DEPs and the small-molecule compounds are shown in Figure 10A. For Hp, the docking score between epinephrine and Hp was the highest. Epinephrine generated hydrogen bonds with GLU314 and LEU334 of Hp (Figure 10B). For COQ9, epinephrine was again the best match. The ASN154, LEU219, ASN252, and GLU255 residues of COQ9 were suggested to be the binding sites of epinephrine (Figure 10C). Furthermore, Tf displayed the strongest binding affinity with sulfadimethoxine, potentially through hydrogen bonding at the LEU294, ARG124, and TYR188+ residues of Tf (Figure 10D). For Prnp, the docking score between chloroform and Prnp was the highest (Figure 10E). Epinephrine was the best match for Acta1, with potential binding at the GLY97, GLY98, and ARG165 residues of Acta1 (Figure 10F). The binding affinity between sulfadimethoxine and Timm44 was the strongest, with potential hydrogen bonding at the ALA330 and TYR421 residues of Timm44 (Figure 10G). Finally, prednisolone acetate was best matched with Abcb6, and ARG739, GLY687, LYS743, and VAL668 of Abcb6 were the potential targets of prednisolone acetate (Figure 10H).

## Discussion

Heart failure with preserved ejection fraction is a complex syndrome that includes many types of clinical phenotypes. Huge pathophysiological differences exist among patients with different clinical HFpEF phenotypes, and no treatment strategy is suitable for all patients with HFpEF (Ge, 2020). Exploring the underlying pathophysiological mechanisms of different types of HFpEF will aid the discovery of personalized therapies and precision medicines for HFpEF treatment. Patients with HFpEF who are also diagnosed with HTN are considered to have vascular-related HFpEF, so exploring the functional connections between HFpEF and HTN will contribute to finding effective therapeutic targets for HFpEF and HTN treatment.

In this study, TMT-labeled quantitative proteomics was used to identify HFpEF- and HTN-related proteins. The functional links between HFpEF and HTN were analyzed at the network, module, and protein levels. Furthermore, molecular docking was used to determine precision medicine targets for HFpEF and HTN treatment. Seven co-DEPs were found among the HFpEF- and HTN-DEPs identified, including Hp, Tf, COQ9, Acat1, Timm44, Abcb6, and Prnp. Notably, Hp levels are closely related to hypertension and heart failure (Schröcksnadel, 1990; Lu et al., 2019; Rodrigues et al., 2019). Moreover, clinical studies have shown that inflammation plays an important role in the transition from HTN to HFpEF (Quaye, 2008), and that Hp is an indicator of inflammation in cardiovascular diseases (Szelényi et al., 2015). This suggests that Hp could be used to diagnose HTN and HFpEF and that Hp could be an effective therapeutic target for HTN and HFpEF. COQ9 is involved in the basic functions of mitochondria (Ferko et al., 2015), and the impairment of mitochondrial function is a common pathophysiological mechanism underlying both HTN and HFpEF (He et al., 2019; Zeng and Chen, 2019). Thus, COQ9 is also a potential biomarker for HTN and HFpEF. Several studies have shown that the expression of Tf and Prnp is altered in many cardiovascular diseases and that Tf and Prnp may be novel biomarkers for HTN and HFpEF (Gao et al., 2013; Rahim et al., 2018; Roura et al., 2018; Pang et al., 2020). Acta1 is involved in the pathological progression of myocardial remodeling (Pagano et al., 2017; Cañes et al., 2020), which is closely related to the prognosis of HTN and HFpEF (Fortuño et al., 2001; Georgiopoulou et al., 2010; Heinzel et al., 2015). Abcb6 and Timm44 are involved in mitochondrial functions and are potential biomarkers for HTN and HFpEF (Boswell-Casteel et al., 2017; Gao et al., 2020). Analysis using the CTD database indicated that there were strong connections between the co-DEPs and cardiovascular diseases in humans, including HF and HTN. Additionally, HFpEF was the most common type of HF, suggesting that the co-DEPs may be effective therapeutic targets for HFpEF and HTN treatment.

Fever and anemia were found to be important co-DEP-related MM symptoms, which is consistent with previous findings (Bocchi et al., 2013; Tanimura et al., 2015; Burns et al., 2018). The common biological processes of HFpEF and HTN were closely related to energy metabolism. Previous studies have also indicated that mitochondrial oxidative capacity plays an important role in both HFpEF and HTN (Gueugneau et al., 2016; De Jong and Lopaschuk, 2017).

Heart failure with preserved ejection fraction and HTN shared eight overlapping modules, and the main biological functions enriched in these modules were myocardial contraction, energy metabolism, apoptosis, oxidative stress, immune response, and cardiac hypertrophy. We also found that post-translational modification and regulation of actin filaments could play an important role in HFpEF and HTN. Previous research has shown that phosphorylation of cardiac myosin-binding protein-C influences the progress of cross-bridge detachment and that deficient phosphorylation leads to diastolic dysfunction (Rosas et al., 2015). Furthermore, fibroblasts with abnormal proliferation contribute to the progression of HTN to HFpEF (Oatmen et al., 2020). Further research into the biological process of barbed-end actin filament capping may provide new insights.

The study also suggested that chloroform, epinephrine, sulfadimethoxine, and prednisolone acetate could be effective drugs for treating HTN and HFpEF. In addition, epinephrine, sulfadimethoxine, and prednisolone acetate have been widely used in many clinical diseases, but chloroform was not approved drug for human use. It suggested that chloroform maybe an candidate drugs for HTN-HFpEF. Previous studies have shown that gut microbiota dysfunction is closely related to the development of HTN and HFpEF (Hsu et al., 2020; Pakhomov and Baugh, 2020), and some antibiotics that regulate the gut microbiota have shown beneficial effects against HTN and other heart diseases (Chen et al., 2020; Du et al., 2020; Wu et al., 2020). A previous study showed that sulfadimethoxine could also regulate the gut microbiota (Mourand et al., 2014) and contribute to the normalization of blood pressure. Notably, chloroform injection can decrease the mean blood pressure (Loyke, 1971). Furthermore, prednisolone can prevent post-transplantation hypertension in rat renal allograft recipients (de Keijzer et al., 1987), indicating that prednisolone may be an effective drug for treating HTN and HFpEF. For patients with mild essential hypertension, intravenous infusion of small amounts of epinephrine has shown beneficial effects on hemodynamics, renal electrolyte excretion, and blood platelets (Kjeldsen et al., 1988). This indicates that epinephrine may be an effective drug for treating HTN and HFpEF. Most treatment strategies for HFpEF are empiric and are greatly influenced by expert consensus. In addition, some treatment strategies showed beneficial effects in patients with HFpEF, including the use of diuretics to control hypervolemia, treatment with mineralocorticoid antagonists, exercise therapies, and classical treatments for comorbidities. The results of this study, which are based on molecular docking and bioinformatics analyses, indicated that chloroform, epinephrine, sulfadimethoxine, and prednisolone acetate could be effective medicines for HTN and HFpEF. These drugs could be used to treat HTN and HFpEF, to reduce the occurrence of HFpEF in patients with HTN, or as personalized medicines for patients with HFpEF. Further animal experiments and small-scale clinical trials are needed to elucidate the functions and effects of these drugs in HTN and HFpEF. Nevertheless, it is important to note that chloroform is currently not approved for human use.

According to the enrichment results of the overlapping modules, myocardial contraction was the most important biological function shared between HFpEF and HTN. HTN influences the structure and function of the heart, suppresses myocardial contractions, and increases the prevalence of HFpEF (Chirinos et al., 2017). Previous studies have also noted the importance of myocardial contraction, as impaired diastolic function is a common phenotype of HFpEF and HTN. Here, the co-DEPs Acat1, Tf, and Hp were associated with myocardial contraction. Acta1 is involved in skeletal muscle thin filament assembly, which influences the contractile force of the heart (Winter et al., 2016). Tf and Hp are related to the response to lead ion, and result in myocardial contraction-related neurotoxic effects (Pappas et al., 2015). Previous studies have indicated that epinephrine treatment enhances myocardial contraction, but the effects of sulfadimethoxine on myocardial contraction remain unclear (Paur et al., 2012).

Here, we found that energy metabolism is closely related to HFpEF and HTN. These findings are consistent with earlier observations (Baltatu et al., 2017; De Jong and Lopaschuk, 2017). Acta1, Timm44, and Abcb6 are involved in fatty acid beta-oxidation and in the biological functions of energy metabolism. In animal experiments, fatty acid beta-oxidation is associated with the severity of myocardial fibrosis. Additionally, it is associated with a risk for HFpEF. Epinephrine, sulfadimethoxine, and prednisolone also have beneficial effects on energy metabolism (Park et al., 2001; Laskewitz et al., 2010; Wang et al., 2019).

Furthermore, apoptosis was found to be an important biological function in HTN and HFpEF. Activation of apoptosis can lead to cardiac dysfunction (Ekhterae et al., 1999), and inhibition of apoptosis can improve heart function and lead to beneficial effects in HFpEF and HTN therapies (Liu et al., 2018; Chen et al., 2019). Hp and Tf were involved in the response to hypoxia, which promotes cardiomyocyte apoptosis. Previous studies have indicated that Prnp is associated with the negative regulation of apoptosis in other diseases (Gao et al., 2019). As chloroform was best matched with Hp, Tf, and Prnp, further studies exploring the anti-apoptotic effect of chloroform in HFpEF and HTN treatment are needed.

In our study, oxidative stress was important in both HFpEF and HTN. Myocardial fibrosis, the major factor leading to myocardial remodeling, was found to be a common pathological mechanism among HFpEF and HTN. Previous studies using an animal model of HFpEF and HTN have confirmed that the regulation of oxidative stress contributes to the inhibition of myocardial fibrosis (Wu et al., 2016; van der Pol et al., 2018). Similarly, other studies showed that Hp and COQ9 are involved in oxidative stress, including cellular oxidant detoxification and negative regulation of oxidoreductase activity (Swain et al., 2020; Yoshida et al., 2020). However, the effect of epinephrine on oxidoreductase activity in HFpEF and HTN still needs to be explored.

Immune responses are activated in both HFpEF and HTN (Carnevale and Wenzel, 2018; Michels da Silva et al., 2019). Although a treatment strategy targeting the immune response achieved some positive results in HTN, no obvious beneficial effects were observed in HFpEF (Michels da Silva et al., 2019; Zhao et al., 2019). Previous studies have shown that aging influences the immune response in HFpEF and HTN, and here, we found that Hp was associated with aging (De la Fuente et al., 2005; Forman and Goodpaster, 2018). Furthermore, Prnp was associated with the negative regulation of the T cell receptor signaling pathway, which is known to influence the immune response (Wong et al., 2017). Thus, chloroform may be an effective drug for targeting the immune response in HFpEF and HTN treatment.

The results showed that cardiac hypertrophy, which is associated with diastolic function, was significantly associated with HFpEF and HTN (Schmieder, 1990). Angiotensin II receptor blockers (ARBs) have been used in clinical trials for the treatment of HTN, as they not only reduce blood pressure but also have beneficial effects on cardiac hypertrophy, diastolic function, and renal function (Israili, 2000). ARBs also affect the blood pressure of patients with HFpEF, but do not have significant effects on echocardiographic parameters, 6-min walk test distances, or brain natriuretic peptide levels (Parthasarathy et al., 2009). Previous studies have shown that epinephrine can also suppress cardiac hypertrophy. Further research is necessary to determine whether chloroform and prednisolone can induce similar beneficial effects in HFpEF and HTN.

## Conclusion

Seven co-DEPs were observed between the HFpEF-DEPs and HTN-DEPs, including Hp, Tf, COQ9, Acat1, Timm44, Abcb6, and Prnp. These co-DEPs were closely related to the main functional similarities of HFpEF and HTN, including myocardial contraction, energy metabolism, apoptosis, oxidative stress, immune response, and cardiac hypertrophy. These co-DEPs may serve as biomarkers and drug targets for HFpEF and HTN. Furthermore, epinephrine, sulfadimethoxine, chloroform, and prednisolone acetate may serve as precision medicines for the treatment of HTN and HFpEF. Our study provides several targets for of the development of personalized therapies and precision medicines to treat HFpEF and other comorbidities.

## Limitations

There are some limitations to this study. Proteins with low expression levels or those showing insignificant changes could have been ignored in the analyses. Furthermore, these results need to be validated through fundamental research and clinical trials. Further animal experiments will help to explore the function of these drugs in HTN and HFpEF, and small-scale clinical trials will contribute to identifying whether these drugs have similar effects in patients with HTN and those with HFpEF.

## Data Availability Statement

The datasets presented in this study can be found in online repositories. The names of the repository/repositories and accession number(s) can be found in the article/Supplementary Material.

## Ethics Statement

The animal study was reviewed and approved by the Animal Ethics Committee of Shandong University of Traditional Chinese Medicine.

## Author Contributions

GZ, JC, and CW conceived the study, acquired the data, and wrote the manuscript. PJ designed the experiments and interpreted the data. YW and YZ performed the experiments and statistical analysis. YJ and XL designed the study and revised the manuscript. All authors read and approved the final manuscript.

## Conflict of Interest

The authors declare that the research was conducted in the absence of any commercial or financial relationships that could be construed as a potential conflict of interest.

## Publisher’s Note

All claims expressed in this article are solely those of the authors and do not necessarily represent those of their affiliated organizations, or those of the publisher, the editors and the reviewers. Any product that may be evaluated in this article, or claim that may be made by its manufacturer, is not guaranteed or endorsed by the publisher.

## Footnotes

1
http://www.uniprot.org

2
http://string-db.org/

3
https://cytoscape.org/

4
https://david.ncifcrf.gov/

5
https://www.cytoscape.org/

6
https://baderlab.org/Software/MCODE

7
http://ctdbase.org/

8
https://www.symmap.org/

9
https://www.drugbank.ca/

10
http://www.rcsb.org/

11
https://pubchem.ncbi.nlm.nih.gov/

12
https://www.symmap.org
